# Enzymatic Hydrolysis Optimization of Yak Whey Protein Concentrates and Bioactivity Evaluation of the Ultrafiltered Peptide Fractions

**DOI:** 10.3390/molecules29061403

**Published:** 2024-03-21

**Authors:** Lingshen Hao, Xuefei Li, Baotang Zhao, Xuemei Song, Yan Zhang, Qi Liang

**Affiliations:** Functional Dairy Products Engineering Laboratory of Gansu Province, College of Food Science and Engineering, Gansu Agricultural University, Anning District, Lanzhou 730070, China; eh81217@163.com (L.H.); 15336010093@163.com (X.L.); zhaobaotang@126.com (B.Z.); songxm@gsau.edu.cn (X.S.); zhangyan@gsau.edu.cn (Y.Z.)

**Keywords:** yak whey protein concentrates, enzymatic hydrolysis optimization, reduce blood sugar, lower uric acid, antioxidant

## Abstract

Yak whey protein concentrates (YWPCs) have good functional properties, but there is still a gap in the study of their peptides. In this study, peptides were obtained by enzymatic hydrolysis, and the bioactivity of each ultrafiltration fraction was evaluated using an optimal process. YWPCs were isolated and purified from yak milk as the raw material. Alkaline protease, trypsin, and papain were used to hydrolyze YWPCs. The protease with the highest degree of hydrolysis (DH) and peptide concentration was selected as the most suitable enzyme. The effects of pH, temperature, time, and the enzyme-to-substrate ratio (E/S) on the DH and peptide concentration were investigated, and response surface methodology was utilized to optimize the hydrolysis process. The hydrolysate was separated using ultrafiltration membranes with molecular weight cut-offs of 10 kDa, 5 kDa, 3 kDa, and 1 kDa. The bioactivity of each ultrafiltration component was analyzed, including the inhibition rates of α-amylase and xanthine oxidase (XOD) activities and the scavenging rates of 2,2′-azino-bis(3-ethylbenzothiazoline-6-sulfonic acid) (ABTS) cation radicals. The results indicated that alkaline protease was the best enzyme for hydrolyzing YWPCs. The peptide concentration in the YWPC hydrolysate was the highest (17.21 mg/mL) at a pH of 8 and a concentration of 7500 U/g, after 2.5 h at 62 °C. The enzymatic hydrolysate was ultrafiltered to yield four peptide fractions, of which the <1 kDa peptides exhibited the highest α-amylase inhibitory activity (22.06%), XOD inhibitory activity (17.15%), and ABTS cationic free radical scavenging rate (69.55%). This demonstrates the potential of YWPC hydrolyzed peptides for hypoglycemic, uric acid-lowering, and antioxidant applications, providing a theoretical basis for the high-value utilization of YWPCs.

## 1. Introduction

Whey is a by-product of cheese and casein production, with the global total output of Holstein whey estimated at approximately 180–190 million tons per year, of which only about 50% is processed, while the remainder is treated as waste effluent [1]. Whey protein can be obtained by concentrating and separating whey liquid, which is primarily composed of alpha-lactalbumin (α-La), beta-lactoglobulin (β-Lg), bovine serum albumin (BSA), lactoferrin (LF), immunoglobulins (Igs) [2], and other components. Whey protein products are mainly categorized into two types: whey protein concentrate (WPC), with a protein content of 34–89%, and whey protein isolate (WPI), with a protein content exceeding 90% [3]. Whey protein is often added to infant formulas; fitness- or athlete-oriented muscle-building powders; nutritional formula powders for the elderly, injured, and sick; and yogurt to provide high-quality protein. Yak (*Bos grunniens*) mainly thrives on the Qinghai–Tibet Plateau in Chinese provinces such as Xinjiang, Gansu, Qinghai, Sichuan, and Tibet, with a population of about 15 million, accounting for over 90% of the global yak numbers [4]. In 2019, China’s annual yak milk production exceeded 800,000 tons, with a protein content of about 4.5–6.5%. However, with few modern industrial processed products being made from it, yak whey has been neglected in terms of recycling and utilization. In our research group’s previous research on the whey expelled from hard yak cheese, it was discovered that the yak whey protein concentrates (YWPCs) obtained through dialysis using a 10,000 Da regenerated cellulose membrane contained no lactose and had a total protein content above 80% and a solution pH greater than 5.1. Its functional properties, including solubility, water-holding capacity, oil-holding capacity, foaming capacity, emulsifying capacity, and thermal stability, were significantly higher than those of samples obtained through dialysis with a 3500 Da roll membrane and a 5000 Da regenerated cellulose membrane. YWPCs have promising prospects for development [5].

Whey proteins are cleaved into peptides using methods such as enzymatic digestion or microbial fermentation. Enzymatic hydrolysis, which is currently the most commonly used method for the preparation of whey protein peptides, is widely used, and its reaction conditions and processes are easy to control. Bioactive peptides typically consist of 2–20 amino acid residues [6]. Studies have shown that bioactive peptides derived from whey protein possess numerous beneficial properties, such as hypoglycemic [7], antioxidant [8], cholesterol-lowering [9], antihypertensive [10], antimicrobial [11], anti-inflammatory [12], and antithrombotic [13] activities, thus promoting human health. Consequently, the production of and research activities regarding yak whey protein peptides is an important means to achieve their high-value utilization.

Currently, the antioxidant, hypoglycemic, and uric acid-lowering functions of bioactive peptides are hotspots for research. Free radicals are generated in the normal metabolic processes of the body, and when the generation and elimination of free radicals in the body are out of balance, oxidative stress occurs, leading to cellular damage, which in turn causes disease [14]. 2,2’-azino-bis(3-ethylbenzothiazoline-6-sulfonicacid) (ABTS) can generate the free radical ABTS+ in the presence of oxidizing agents, so scavenging ABTS cationic radicals has an antioxidant effect. Diabetes mellitus is a chronic metabolic disease, the most common form of which is type II diabetes mellitus, which accounts for about 90–95% of all cases [15]. α-amylase breaks down polysaccharide molecules into monosaccharides that are more easily digested and absorbed, leading to postprandial hyperglycemia and elevated blood glucose levels, and the inhibition of α-amylase activity helps to reduce postprandial blood glucose levels, useful for preventing and treating type II diabetes mellitus [16]. Hyperuricemia is a metabolic disease caused by the chaotic metabolism of purine-like substances or reduced excretion of uric acid. Xanthine oxidase (XOD) reacts with xanthine, yielding uric acid, and the inhibition of XOD activity helps to reduce uric acid levels, thus helping to combat hyperuricemia.

To date, there has been no research on the preparation of peptides from YWPCs nor on the effects of these peptides following ultrafiltration fractionation on the inhibition of α-amylase and XOD activities, nor on their scavenging effects on ABTS radical cations. Consequently, in this experiment, we first subjected YWPCs to enzymatic hydrolysis to select the most appropriate enzyme. Peptide concentration was used as the response value to optimize the hydrolysis conditions for YWPCs via the response surface method. Enzymatic hydrolysate was separated using four different molecular weight cut-off ultrafiltration membranes, followed by lyophilization into powder. The α-amylase and XOD activity inhibition rates, along with the ABTS radical cation scavenging rates of different peptide fractions, were analyzed.

## 2. Results

### 2.1. The Effects of Different Enzymes on the Degree of Hydrolysis (DH) and Peptide Concentration of YWPCs

The DH represents the percentage of free amino nitrogen in a protein hydrolysate relative to the total nitrogen content, indicating the degree of peptide fragmentation. The fundamental purpose of hydrolyzing proteins is to control the degree of protein hydrolysis, minimize the production of free amino acids, and maximize the yield of target polypeptides. Therefore, the effects of different proteases on YWPCs (with an 80% protein content) are evaluated based on the indicators of the DH and peptide concentration.

**Figure 1:**
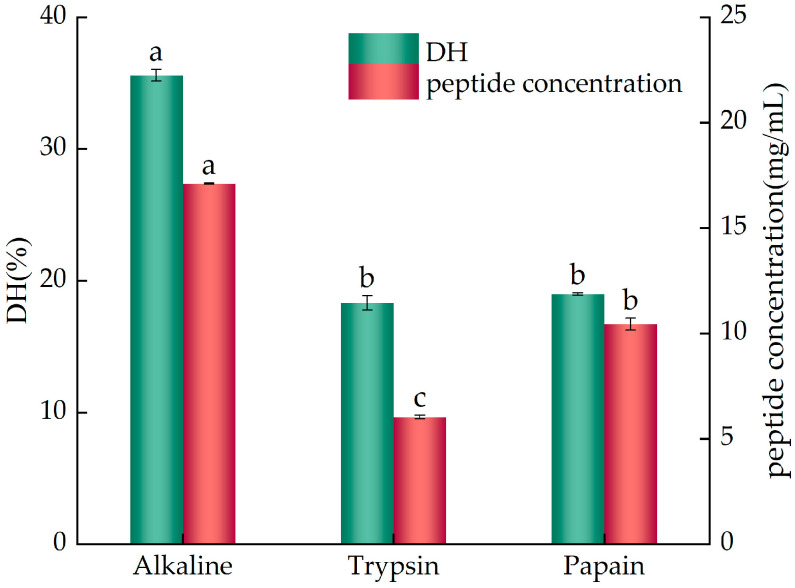
The effects of different proteases on the DH and peptide concentration of YWPCs. Different letters indicate significant differences between samples (*p* < 0.05).

As shown in Figure 1, under the action of different enzymes, differences in the DH and peptide concentration of YWPCs were observed. The DH of the alkaline protease hydrolysate was 35.59%, which was significantly higher than that of the trypsin hydrolysate (18.33%) and papain hydrolysate (19.01%) (*p* < 0.05), but there was no significant difference in the DH between the trypsin hydrolysate and papain hydrolysate (*p* > 0.05). The peptide concentration of the alkaline protease hydrolysate was 17.11 mg/mL, which was also significantly higher than that of the trypsin hydrolysate (6.04 mg/mL) and papain hydrolysate (10.45 mg/mL) (*p* < 0.05), and there was a significant difference in peptide concentrations between the trypsin and papain hydrolysates (*p* < 0.05). Therefore, alkaline protease was selected as the enzyme for this experiment, which ensured an appropriate DH while enhancing the peptide concentration of the hydrolysate.

### 2.2. Single-Factor Experimental Results and Analysis

#### 2.2.1. The Influence of pH and Temperature on the Enzymatic Hydrolysis of YWPCs

**Figure 2:**
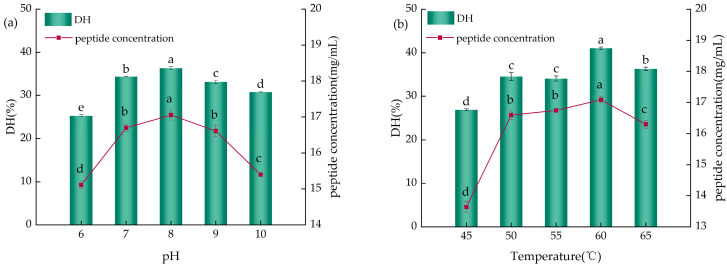
(**a**) The influence of different pH levels on the DH of YWPCs and the concentration of peptides. (**b**) The influence of different temperatures on the DH of YWPCs and the concentration of peptides. Different letters indicate significant differences between samples (*p* < 0.05).

As depicted in Figure 2a, with the increase in pH, both the DH and peptide concentration of the YWPC enzymatic hydrolysate showed a trend of an increase followed by a decrease, reaching their peak values at pH 8, with the DH (36.29%) and peptide concentration (17.05 mg/mL) being significantly higher compared to the other levels (*p* < 0.05), indicating that at pH 8, the enzyme’s spatial structure is most favorable, its activity is optimal, and it can bind more efficiently with YWPCs; therefore, pH 8 was chosen as the most suitable pH for the reaction.

As shown in Figure 2b, as the temperature increased, both the DH and peptide concentration of the YWPCs enzymatic hydrolysate overall presented a trend of an increase followed by a decrease, and at 60 °C, the values for the DH (41.05%) and peptide concentration (17.09 mg/mL) reached their maxima, significantly exceeding the other levels (*p* < 0.05), demonstrating that at a reaction temperature of 60 °C, the enzymatic activity was fully utilized, a result consistent with that reported by Ji et al. [17], who also found that an increase in temperature is conducive to hydrolysis. Thus, 60 °C was selected as the most appropriate reaction temperature.

#### 2.2.2. The Impact of Time and The Enzyme-to-Substrate Ratio (E/S) on the Enzymatic Hydrolysis of YWPCs

**Figure 3:**
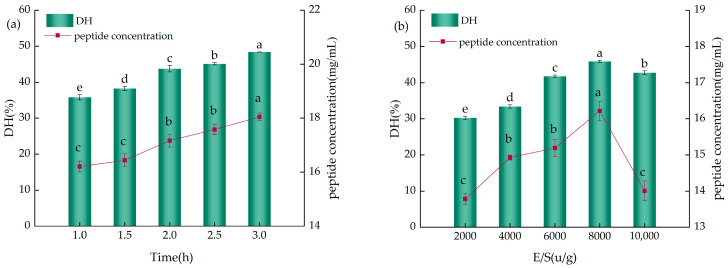
(**a**) The influence of different times on the DH and peptide concentration of YWPCs. (**b**) The influence of different E/S ratios on the DH and peptide concentration of YWPCs. Different letters indicate significant differences between samples (*p* < 0.05).

As shown in Figure 3a, with the increase in hydrolysis time, both the DH and peptide concentration of the enzymatic hydrolysate exhibited an upward trend; however, the rates of increase varied across different time intervals. When hydrolysis time increased from 1 h to 2 h, the DH of the hydrolysate increased by 7.93%, and the peptide concentration increased by 0.96 mg/mL, indicating a more pronounced growth trend, whereas when hydrolysis time was extended from 2 h to 3 h, the DH of the hydrolysate and the peptide concentration increased by 4.64% and 0.87 mg/mL, respectively, indicating a decelerated growth trend. Sun et al. [18] also indicated that the DH value increases rapidly during the first 2 h of the alkaline protease substrate reaction and then rises more slowly. Moreover, research shows that during the initial 10 min of hydrolysis with alkaline protease, pH rapidly decreases, necessitating continuous NaOH addition to maintain optimal pH for alkaline protease reaction, which can lead to a decline in the quality and flavor of the hydrolysate as well as large accumulations of NaCl [19]. Continuous extension of hydrolysis time does not result in clear inflection points in the DH or peptide concentrations; therefore, considering both time and economic efficiency, a hydrolysis duration of 2 h was chosen as the reaction time.

As indicated in Figure 3b, with the increase in the E/S ratio, the DH and peptide concentration of the YWPC enzymatic hydrolysate initially rose and then fell, exhibiting significant differences (*p* < 0.05). When the E/S ratio ranged from 2000 U/g to 8000 U/g, the DH of the hydrolysate increased by 15.64%, and the peptide concentration rose by 2.43 mg/mL, but at E/S ratios ranging from 8000 U/g to 10,000 U/g, the hydrolysate’s DH decreased by 3.11%, and peptide concentration decreased by 2.21 mg/mL. This result suggests that at an E/S ratio of 8000 U/g, the enzyme binds more effectively with YWPC, and studies by Ji et al. [17] also indicate that an increase in enzyme concentration positively affects protein hydrolysis; hence, an E/S ratio of 8000 U/g was chosen as the optimal ratio.

### 2.3. Analysis of the Results from the Response Surface Optimization Experiment

#### 2.3.1. Results of the Response Surface Optimization Experiment and Analysis of Variance for the Regression Equation

On the basis of single-factor experiments, three parameters with a significant influence on YWPC enzymatic hydrolysis were selected: temperature (A), time (B), and E/S ratios (C). A three-factor, three-level response surface methodology optimization experiment was designed, with the hydrolysate’s peptide concentration (mg/mL) being used as the response variable to determine the optimal enzymatic hydrolysis conditions for YWPCs. Table 1 displays the response surface experiment design and results, while Table 2 presents the analysis of variance for the response surface experiment data.

Multivariate regression fitting was conducted on the results in Table 1 using Design-Expert 12 statistical analysis software, resulting in a second-order regression equation for peptide concentration (Y) as a function of temperature (A), time (B), and E/S ratios (C): Y = 15.87 + 1.53A + 0.6150B − 0.6563C + 0.6725AB + 0.5900AC − 0.0175BC − 2.60A^2^ + 0.3445B^2^ − 1.72C^2^.

As shown in Table 2, the model’s *p*-value of <0.0001 indicates that the regression model is highly significant. The lack-of-fit term reflects the degree of fit between the model and the experiment, that is, the disparity between them. In this case, the lack-of-fit *p*-value of 0.2321 > 0.05 is not significant, suggesting the model is highly reliable; the determination coefficient R^2^ = 0.9763 indicates a good fit between the experimental and predicted values, while the adjusted determination coefficient R^2^_Adj_ = 0.9457 suggests that 94.57% of the variance in the response values can be explained by the model. The F-value indicates the order of the factors affecting the response values in terms of precedence: temperature (A) > E/S ratio (C) > time (B). The primary terms A and C and the quadratic terms A^2^ and C^2^ have a highly significant effect on peptide concentration, whereas the primary term B and the interaction terms AB and AC have a significant impact. Therefore, this model can be used for analyzing the peptide concentration of YWPC enzymatic hydrolysates.

#### 2.3.2. Response Surface Analysis

**Figure 4:**
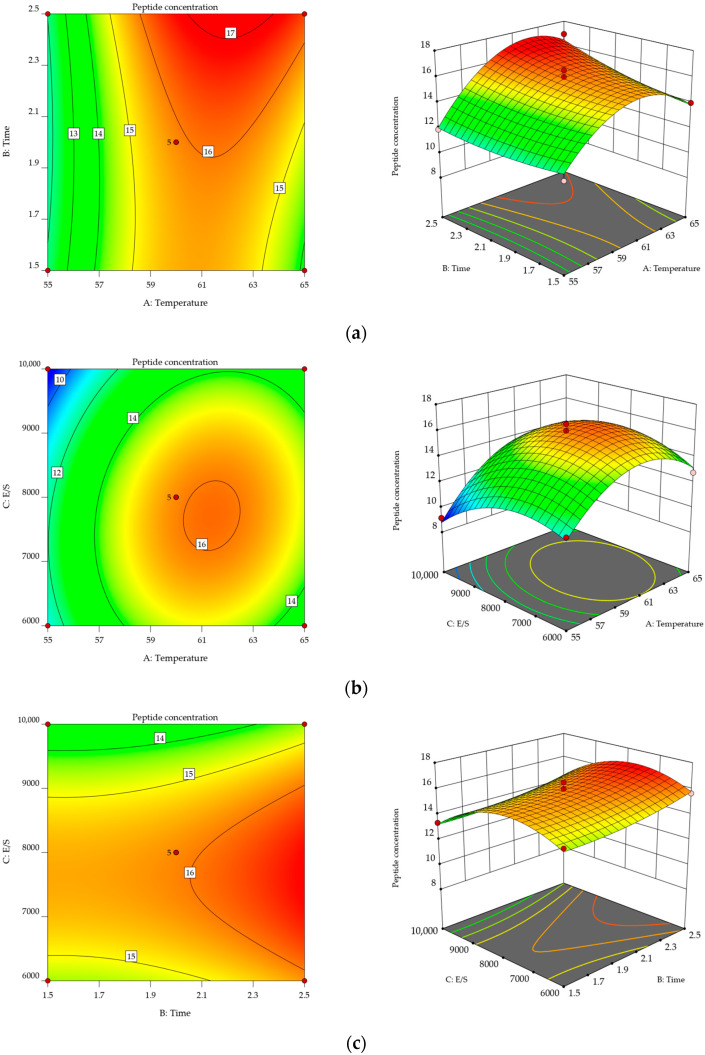
Results of the response surface experiment. (**a**) Response surface and contour plot of the effects of reaction temperature and reaction time on peptide concentration. (**b**) Response surface and contour plot of the effects of reaction temperature and E/S ratio on peptide concentration. (**c**) Response surface and contour plot of the effects of reaction time and E/S ratio on peptide concentration.

Figure 4 illustrates response surface plots of the interactions among factors affecting peptide concentration. Figure 4a, Figure 4b, and Figure 4c each represent the impact of the interaction between temperature and time (AB), temperature and E/S ratio (AC), and time and E/S ratio (BC) on the peptide concentration of the YWPC enzymatic hydrolysate, respectively. Response surface plots can accurately reflect the interactions between different factors and their impact on the response values. The smoother the slope of the response surface, the smaller the effect of the factor’s variation on the response value. Conversely, a steeper slope indicates a larger effect of the factor’s variation on the response value [20].

The optimal conditions determined via response surface software analysis are a temperature of 61.603 °C, a time of 2.494 h, and an E/S ratio of 7492.245 U/g, resulting in a peptide concentration of 17.258 mg/mL. For operational convenience, the conditions were modified to a temperature of 62 °C, a time of 2.5 h, and an E/S ratio of 7500 U/g. The measured peptide concentration was 17.21 mg/mL, which is close to the predicted value.

### 2.4. α-Amylase-Inhibitory Activity of the Ultrafiltration Fractions of YWPC Enzymatic Hydrolysate

**Figure 5:**
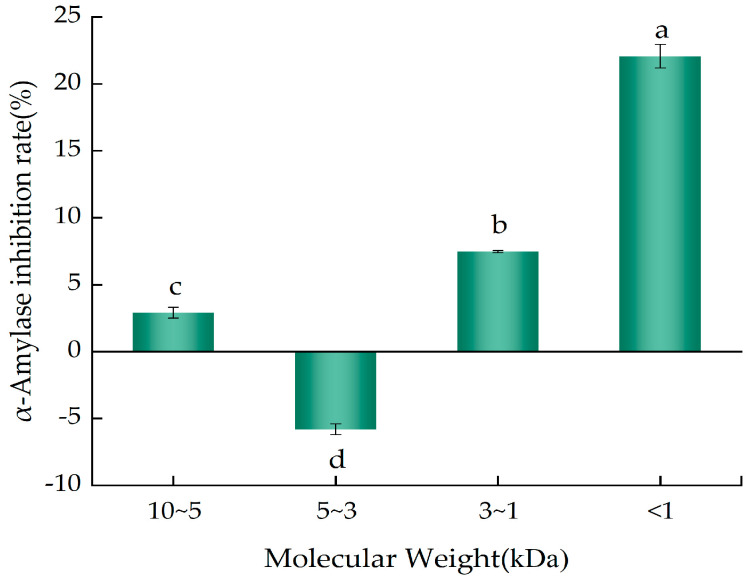
α−amylase inhibition rates of different ultrafiltration fractions of YWPC peptides. Different letters indicate significant differences between samples (*p* < 0.05).

The YWPC enzymatic hydrolysate with the highest peptide concentration was ultrafiltered through membranes with nominal molecular weight cut-offs of 10 kDa, 5 kDa, 3 kDa, and 1 kDa, yielding four peptide fractions with different molecular weight ranges: 10~5 kDa, 5~3 kDa, 3~1 kDa, and <1 kDa. Figure 5 presents the α-amylase inhibition percentages of the components from different molecular weight ranges at the same concentration (10 mg/mL). All three fractions inhibited α-amylase activity with significant differences (*p* < 0.05), with peptides of <1 kDa having the highest inhibition rate (22.06%), which was significantly greater than that of the 3~1 kDa peptides (7.47%) (*p* < 0.05). This indicates that peptides of <1 kDa in the YWPC enzymatic hydrolysate are more effective in inhibiting α-amylase activity, suggesting their potential for lowering blood sugar levels. It is speculated that the reason for this difference in inhibition rates may be due to the fact that some substance has a higher molecular weight, is less hydrophobic, and has fewer amino acid residues (Leu, Pro, and Phe) [21] with the potential to inhibit the activity of α-amylase, making it difficult for them to bind to the active center of α-amylase, resulting in a weaker or non-existent inhibitory effect.

### 2.5. XOD-Inhibitory Activity of the Ultrafiltration Fractions of YWPC Enzymatic Hydrolysate

**Figure 6:**
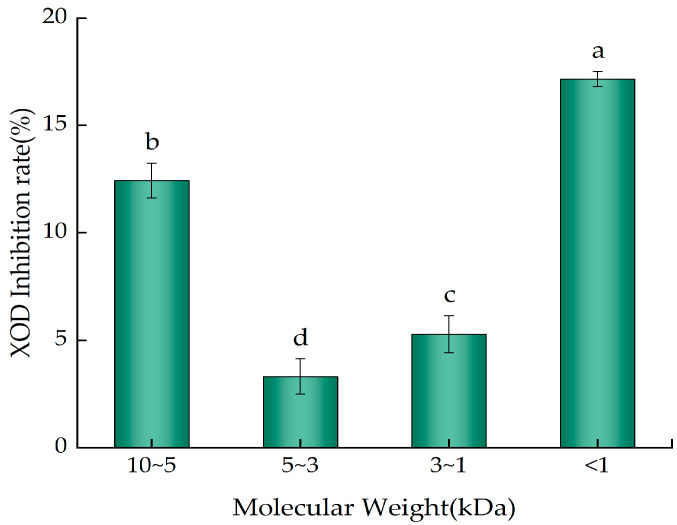
XOD inhibition rate of different ultrafiltration fractions of YWPC peptides. Different letters indicate significant differences between samples (*p* < 0.05).

Figure 6 illustrates the inhibition percentages of XOD activity by components with different molecular weight ranges at the same concentration (10 mg/mL). All four fractions exhibited inhibitory activity against XOD with significant differences (*p* < 0.05); the <1 kDa YWPC peptides showed the highest inhibition rate (17.15%), significantly surpassing those of 10~5 kDa peptides (12.43%), 5~3 kDa peptides (3.31%), and 3~1 kDa peptides (5.27%) (*p* < 0.05). This result suggests that the <1 kDa peptides in the YWPC enzymatic hydrolysate are more capable of inhibiting XOD activity, indicating their potential for reducing uric acid levels. Li et al. [22] found that peptides containing tryptophan (Trp) can effectively inhibit XOD activity, and an increase in the number of Trp residues significantly enhances XOD-inhibitory activity, suggesting that the differences in inhibition rates among the fractions may be attributed to the varying amounts of Trp.

### 2.6. ABTS Cation Radical Scavenging Ability of the Ultrafiltration Fractions of YWPC Enzymatic Hydrolysate

**Figure 7:**
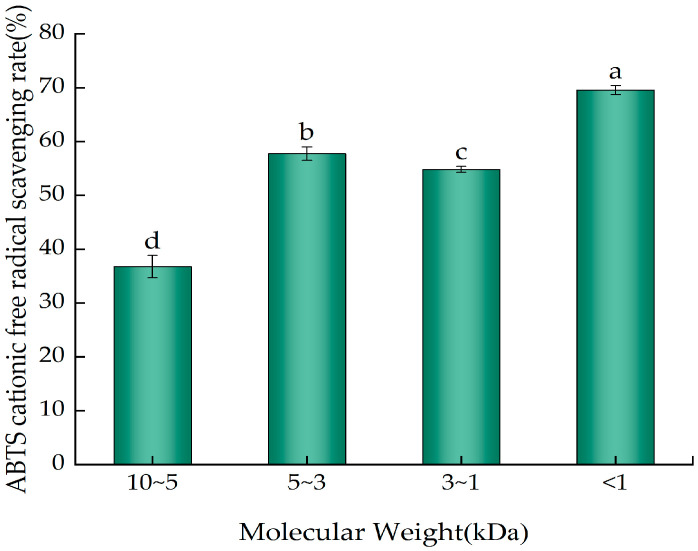
ABTS cation radical scavenging rates of different ultrafiltration fractions of YWPC peptides. Different letters indicate significant differences between samples (*p* < 0.05).

Figure 7 depicts the scavenging rates of ABTS cation radicals by components from different molecular weight ranges at the same concentration (0.2 mg/mL). All four fractions demonstrated the ability to scavenge ABTS radicals, for which significant differences were observed (*p* < 0.05). The peptides <1 kDa had the highest ABTS cation radical scavenging rate (69.55%), followed by the 5~3 kDa peptides (57.75%) and the 3~1 kDa peptides (54.81%), with the 10~5 kDa peptides showing the lowest rate (36.76%). These results indicate that the <1 kDa YWPC peptides are more capable of scavenging ABTS cation radicals, suggesting their potential antioxidant capacities.

## 3. Discussion

This study focuses on the high-value utilization of YWPCs, wherein the enzymatic hydrolysis of YWPCs can enhance functional properties without affecting nutritional value [23]. Three alkaline proteases (alkaline protease, papain, and trypsin) were used to hydrolyze YWPCs, and the results indicated that alkaline protease was the most suitable enzyme, yielding the highest DH and peptide concentration in its hydrolysate. This result was attributed to the different active sites that various proteases have on the substrate YWPCs; enzymes with a greater number of active sites tend to result in higher efficiency of hydrolysis and DH. Alkaline protease is an endopeptidase with extensive specificity, catalyzing cleavage sites on the carboxyl side of aromatic and hydrophobic amino acids [24]. Papain exhibits broad specificity during prolonged incubation, and it is capable of cleaving peptide bonds in hydrophobic regions, including those involving Phe, Tyr, Leu, Ile, Ala, Trp, and Val [25]. The active sites of trypsin are at the carboxyl end of Arg or Lys. Du et al. [26] investigated the effects of seven proteases on the DH of whey protein, also finding that the hydrolysates from alkaline protease had the highest DH. Liu et al. [27] utilized five proteases to hydrolyze mung bean protein and similarly demonstrated that alkaline protease yielded the highest DH.

Enzymatic hydrolysis is influenced by factors such as pH, temperature, time, and E/S ratio, which affect the extent of the reaction. pH affects enzyme stability and the maintenance of non-covalent bonds, altering the spatial conformation and three-dimensional structure of the enzyme molecule, potentially leading to denaturation or inactivation [28]. The temperature of enzymatic hydrolysis impacts enzyme stability and reaction rate; within the optimal temperature range, there is an increase in activated molecules, yielding the highest enzyme activity and reaction speed. At the onset of enzymatic hydrolysis, when enzymes and substrates are in abundance, the reaction proceeds rapidly, with a swift increase in the DH and peptide concentration. Over time, substrates and proteases are gradually consumed [29], protease activity decreases, and the relative content of peptides and free amino acids in the product increases, slowing down the reaction as it becomes inhibited. When the E/S ratio is low, a higher concentration of substrate accumulates at the enzyme’s active site, preventing full enzyme–substrate interaction and resulting in a slower hydrolysis rate. As the E/S ratio increases, the substrate is dispersed more uniformly around the enzyme, improving their interaction and enhancing the rate of hydrolysis. A further increase in the E/S ratio decreases the substrate-to-enzyme ratio, and with limited substrate availability, excess enzymes can lead to excessive protein hydrolysis, breaking down peptide chains and generating free amino acids, consequently reducing peptide concentrations. Therefore, in this study, we optimized the enzymatic hydrolysis conditions for YWPCs via single-factor and response surface methodology experiments. The optimized results indicate that the hydrolysis pH (8) and temperature (62 °C) are consistent with the enzyme’s characteristics, with a moderate hydrolysis time (2.5 h) and E/S ratio (7500 U/g). Al-Bukhaiti et al. [30] enzymatically hydrolyzed peanut protein using alkaline protease and found the optimal conditions to be a pH of 8.41, a temperature of 56.18 °C, an E/S ratio of 6%, and a duration of 3 h.

Peptides with molecular weights <1 kDa in YWPC hydrolysates exhibited the highest inhibitory rates against α-amylase and XOD, as well as the highest clearance rates of ABTS cationic free radicals, suggesting that these low-molecular-weight peptides possess better hydrophobicity and exhibit stronger bioactivity, in agreement with previous studies. Rubén [31] showed that molecular weight is one of the important factors affecting the physiological activity of peptides, including their inhibitory activity against α-amylase, with smaller peptides having a stronger ability to inhibit α-amylase activity. Deng et al. [32] obtained <3 kDa peptide components from hydrolyzed products that demonstrated significant hypoglycemic potential. Through computational simulations, three novel peptides with hypoglycemic abilities were identified, with the largest one having a molecular weight of 1101.32 Da. Li et al. [33] used walnut powder as a raw material and, through enzymatic hydrolysis, isolation, purification, and characterization, identified two peptides with molecular weights less than 1000 Da, both capable of preventing substrates from entering the hydrophobic channel of XOD and exhibiting strong in vitro inhibitory activity against XOD. He et al. [34] isolated and identified 13 dipeptides and tripeptides with XOD-inhibitory activity from hydrolyzed tuna, all with molecular weights <1000 Da. Hamzeh et al. [35] enzymatically hydrolyzed cuttlefish mantles using alkaline protease and compared the antioxidant activity of four fractions separated via ultrafiltration with unseparated hydrolysates, finding that peptides with molecular weights <3000 Da possessed the strongest capacity to scavenge ABTS cationic free radicals. Liu et al. [36] hydrolyzed yak casein using alkaline protease and trypsin and found that the <3 kDa fraction post-ultrafiltration had the highest antioxidant activity at 5.0 mg/mL.

Additionally, further in-depth research on YWPC bioactive peptides is required, primarily focusing on investigating the sequences of peptides with molecular weights <1 kDa; analyzing the physicochemical properties of peptides using bioinformatics tools; elucidating the mechanisms of action of inhibitory peptides through molecular docking techniques; and conducting in vitro activity validation, among other topics.

## 4. Materials and Methods

### 4.1. Materials and Reagents

Yak milk was sourced from Jili Village in Gannan Tibetan Autonomous Prefecture, Gansu Province; transported to our laboratory in a thermostatic container (4 ± 2 °C); and stored at 4 °C to retain spare parts. Alkaline protease (20,000 U/g) (EC.3.4.21.14), o-phthalaldehyde (OPA), xanthine oxidase (XOD), and trypsin (250,000 U/g) (EC.3.4.21.4) were purchased from Shanghai YuanYe Bio-Technology Co., Ltd. (Shanghai, China). Papain (1,200,000 U/g) (EC.3.4.22.2) was purchased from Beijing Coolaber Technology Co., Ltd. (Beijing, China). Alpha-amylase and 3,5-dinitrosalicylic acid (DNS) were purchased from Shanghai Macklin Biochemical Co., Ltd. (Shanghai, China). The 10 kDa and 3 kDa ultrafiltration centrifuge tubes were purchased from Merck Millipore Co., Ltd. (Burlington, MA, USA). The 5 kDa ultrafiltration centrifuge tube was purchased from Sartorius Co., Ltd. (Göttingen, Germany). The 1 kDa ultrafiltration centrifuge tube was purchased from Pall Co., Ltd. (Show Low, AZ, USA). Other chemicals and reagents were of analytical grade and commercially available.

### 4.2. Preparation of YWPCs

YWPCs were prepared using the method reported by Gao Ruiping et al. [5]. Yak whey liquid was collected from the process of hard cheese production in the Functional Dairy Product Engineering Laboratory of Gansu Province and dialyzed for 48 h with a dialysis bag with a molecular weight cut-off of 14,000 Da, and the retentate was lyophilized to obtain YWPCs, which were then stored at 4 °C for later use.

### 4.3. Preparation of YWPC Hydrolysate

YWPC powder was dissolved in deionized water to obtain a 40 mg/mL solution. The pH and temperature of the solution were adjusted to the appropriate conditions for enzymatic hydrolysis with protease. The solution was then stirred constantly at a controlled temperature using a heating magnetic stirrer. Protease was added at an E/S ratio of 6000 U/g, and the pH was maintained by the continual addition of 1 mol/L of NaOH solution. After 1 h of hydrolysis, the enzymes were inactivated via a boiling water bath for 10 min, followed by cooling to room temperature. The mixture was then centrifuged at 5000 rpm for 15 min, and the supernatant was collected.

### 4.4. Optimization of YWPC Hydrolysis Process

#### 4.4.1. Selection of Proteases

The method in Section 4.3 was used to enzymatically hydrolyze YWPCs at the appropriate temperature and pH with papain, trypsin, and alkaline protease. The hydrolysis conditions for different proteases are shown in Table 3. The DH and the concentration of peptides in the hydrolysate were measured to select the most suitable protease for hydrolyzing YWPCs.

#### 4.4.2. Single-Factor Experimental Design

Using alkaline protease as the most suitable protease, the mass concentration used for the YWPC solution was 40 mg/mL. The hydrolysis conditions were as follows: pH 8, a temperature of 55 °C, a duration of 1.0 h, and an E/S ratio of 6000 U/g. Single-factor experiments were carried out, using pH levels (6, 7, 8, 9, and 10), temperature (45, 50, 55, 60, and 65 °C), time (1.0, 1.5, 2.0, 2.5, and 3.0 h), and enzyme-to-substrate ratios (2000, 4000, 6000, 8000, and 10,000 U/g) as the variables. When optimizing one variable, the values of the other variables were kept constant. The hydrolysis parameters were selected based on a comprehensive consideration of the DH and peptide concentration.

#### 4.4.3. Response Surface Optimization

The hydrolyzing enzyme used was alkaline protease, with a reaction pH of 8. Based on the single-factor optimization experiments, the enzymatic hydrolysis temperature, time, and E/S of the protease were used as independent variables, with peptide concentration serving as the response value. Response surface analysis was conducted using Design-Expert 12 software, and a three-factor, three-level response surface optimization experiment was performed, as shown in Table 4.

### 4.5. Measurement of DH

The DH of whey protein was measured using the OPA method, referring to the work of Nielsen et al. [37], with appropriate modifications. The steps of the measurement process are as follows. Precisely withdraw 100 μL from the enzymatic hydrolysate and dilute it to an appropriate factor. Thereafter, take 400 μL of the diluted sample and add it to 3 mL of OPA solution. After thoroughly mixing and reacting for 2 min, the absorbance at 340 nm was measured. A standard curve was plotted using serine as the standard, with distilled water serving as a blank. The relationship between the absorbance value y and serine concentration (mmol/L) x was expressed as y = 0.8259x + 0.0534, with R^2^ = 0.9997, and DH was calculated according to Formula (1).

(1) DH%=(C×N×Vm−β)αHtot

where C (mmol/L) represents the concentration of the sample obtained from the standard curve, N is the dilution factor of the hydrolysate, V (L) is the volume of the sample hydrolysate, M (g) denotes the mass of protein in the sample (for whey protein, α = 1.00 and β = 0.40), and H_tot_ is the number of millimoles of peptide bonds per gram of raw protein (for whey protein, H_tot_ = 8.8 mmol/g).

### 4.6. Peptide Concentration Determination

The method proposed by Xing et al. [38] was employed, with appropriate modifications. The enzymatic hydrolysate was mixed with an equal volume of 10% trichloroacetic acid and allowed to stand for 10 min, followed by centrifugation at 4000 r/min for 10 min. The supernatant was diluted appropriately, and 1 mL of the diluted supernatant was mixed with 4 mL of biuret reagent; then, the mixture was left in the dark for 30 min, and absorbance was measured at a 540 nm wavelength. Distilled water served as the blank. A standard curve was then drawn using bovine serum albumin as the standard, with the relationship between the absorbance (y) and the concentration of bovine serum albumin (mg/mL) (x) defined as y = 0.0543x + 0.0837, with R^2^ = 0.9988. The peptide concentration in the hydrolysate was calculated against the standard curve.

### 4.7. Ultrafiltration Purification of YWPCs’ Enzymatic Products

The enzymatic hydrolysate was subjected to ultrafiltration separation using ultrafiltration centrifuge tubes with molecular weight cut-offs of 10 kDa, 5 kDa, 3 kDa, and 1 kDa and centrifuged at 4000 r/min for 10 min, with this process being repeated multiple times, and both the retentates and filtrates were collected to obtain peptides of different molecular weight ranges. Each fraction was then freeze-dried and further analyzed for its activity.

### 4.8. Determination of α-Amylase-Inhibitory Activity

The method reported by Apostolidis et al. [39] was adopted to measure the α-amylase-inhibitory activity, with some modifications. A 100 μL aliquot of the sample solution was mixed with 100 μL of a 5.2 U/mL α-amylase solution and incubated at 37 °C for 10 min. Then, 100 μL of 1% soluble starch solution was added, followed by another incubation at 37 °C for 10 min. Subsequently, 2 mL of DNS reagent was added, and the mixture was placed in a boiling water bath for 10 min. After cooling to room temperature, 2 mL of distilled water was added to dilute the mixture. Absorbance was measured at 540 nm. The α-amylase inhibition rate was calculated using Equation (2).

(2) $$\alpha -\mathrm{amylase}-\mathrm{inhibitory}\;\mathrm{activity}\;(\%)=\left(1-\frac{A_{0}-A_{1}}{A_{2}-A_{3}}\right)\times 100$$

where A_0_ is the absorbance of the sample group, A_1_ is the absorbance of the sample blank group, A_2_ is the absorbance of the control group, and A_3_ is the absorbance of the blank group.

### 4.9. Determination of XOD-Inhibitory Activity

The method described by Umamaheswari et al. [40] was employed, with some modifications, to determine the inhibitory activity toward XOD. A total of 100 μL of sample solution was mixed with 400 μL of phosphate buffer (pH 7.5) and 100 μL of a 0.2 U/mL XOD solution and incubated at 25 °C for 15 min. Then, 1 mL of xanthine solution was added, followed by a further incubation at 25 °C for 30 min. Subsequently, 100 μL of 1 mol/L HCl was added. The absorbance was measured at a wavelength of 290 nm. The inhibition rate for XOD activity was calculated using Equation (3).

(3) $$\mathrm{XOD}-i\mathrm{nhibitory}\;a\mathrm{ctivity}\;(\%)=\left(1-\frac{A_{1}-A_{2}}{A_{3}-A_{4}}\right)\times 100$$

where A_1_ is the absorbance of the sample group, A_2_ is the absorbance of the sample blank group, A_3_ is the absorbance of the control group, and A_4_ is the absorbance of the blank group.

### 4.10. ABTS Cation Radical Scavenging Ability Determination

The method reported by Ngoh et al. [21] was followed, with necessary adjustments, to assess the ABTS cation radical scavenging activity. A 7 mmol/L solution of ABTS was mixed with a 2.45 mmol/L potassium persulfate solution in equal volumes and left in the dark for 16 h to prepare the ABTS stock solution. The stock solution was diluted with a phosphate buffer solution (pH 7.4) to achieve an absorbance of 0.70 ± 0.02 at 734 nm. Then, 200 μL of the sample solution and 1800 μL of the ABTS solution were thoroughly mixed and incubated in the dark at 30 °C for 10 min. The absorbance was measured at 734 nm. The scavenging rate of ABTS cation radicals was calculated using Equation (4).

(4) $$\mathrm{ABTS}\;\mathrm{cationic}\;\mathrm{radical}\;\mathrm{scavenging}\;\mathrm{rate}\;(\%)=\left(\frac{A_{1}-A_{2}}{A_{1}}\right)\times 100$$

where A_1_ is the absorbance of the control, and A_2_ is the absorbance of the sample.

### 4.11. Data Processing

Each treatment was replicated three times. Data were analyzed for significant differences using SPSS Statistics 26.0 software, and figures were generated using Origin 2021 software.

## 5. Conclusions

In this study, yak milk was used as a raw material to obtain YWPCs through techniques such as separation, purification, and lyophilization. Peptides were prepared using proteolytic hydrolysis, and proteases suitable for hydrolyzing YWPCs were screened. A response surface methodology was applied to optimize the hydrolysis conditions. The optimized YWPC hydrolysate was purified via ultrafiltration, yielding four peptide fractions with relative molecular weights of 10~5 kDa, 5~3 kDa, 3~1 kDa, and <1 kDa. The inhibitory rates toward α-amylase and XOD activity and the scavenging rates of ABTS cationic free radicals were analyzed for different fractions. The results indicated that alkaline protease was the best enzyme for preparing YWPC peptides. The optimal hydrolysis conditions obtained through response surface methodology optimization were pH 8, 62 °C, 2.5 h, and 7500 U/g. Under these conditions, the peptide concentration of the YWPC hydrolysate was 17.21 mg/mL, which was close to the predicted value. Additionally, this study found that the molecular weight of YWPC peptides significantly influences their bioactive characterization; in particular, peptides with a <1 kDa molecular weight showed the highest α-amylase inhibition (22.06%), XOD-inhibitory rate (17.15%), and ABTS cationic free radical scavenging rate (69.55%), indicating that the low-molecular-weight peptides in the YWPC hydrolysate possess good hypoglycemic, uric-acid-reducing, and antioxidant capabilities. This study provides a theoretical basis for the high-value utilization of YWPCs, the development of bioactive peptides, and the study of the corresponding active mechanisms. Subsequent work can include the mass-spectrometric identification of YWPC low-molecular-weight peptides and further analysis of their physicochemical properties and inhibitory mechanisms.

## Figures and Tables

**Table 1:** The response surface experiment design and results.

Run	A Temperature (°C)	B Time (h)	C E/S (U/g)	Polypeptide Concentration (mg/mL)
1	60	2	8000	15.46
2	60	2	8000	15.68
3	65	1.5	8000	13.99
4	60	1.5	10,000	13.36
5	65	2.5	8000	16.92
6	55	2.5	8000	11.89
7	55	2	6000	11.53
8	60	2	8000	16.01
9	60	2	8000	16.50
10	55	1.5	8000	11.65
11	55	2	10,000	9.15
12	65	2	10,000	12.75
13	60	2.5	10,000	14.20
14	60	1.5	6000	14.75
15	65	2	6000	12.77
16	60	2.5	6000	15.66
17	60	2	8000	15.68

**Table 2:** The analysis of variance for the response surface experiment data.

Source	Sum of Squares	Degrees of Freedom	Mean Square	F-Value	*p*-Value	Significance
Model	71.30	9	7.92	31.98	<0.0001	Significant
A-Temperature	18.64	1	18.64	75.22	<0.0001	**
B-Time	3.03	1	3.03	12.21	0.0101	*
C-E/S	3.45	1	3.45	13.91	0.0074	**
AB	1.81	1	1.81	7.30	0.0305	*
AC	1.39	1	1.39	5.62	0.0496	*
BC	0.0012	1	0.0012	0.0049	0.9459	
A^2^	28.42	1	28.42	114.71	<0.0001	**
B^2^	0.4997	1	0.4997	2.02	0.1985	
C^2^	12.43	1	12.43	50.16	0.0002	**
Residual	1.73	7	0.2477			
Lack of fit	1.08	3	0.3592	2.19	0.2321	no Significant
Pure error	0.6567	4	0.1642			
Cor total	73.03	16				
R^2^ = 0.9763			R^2^_Adj_ = 0.9457			

Note: * indicates a significant difference (*p* < 0.05), ** indicates a very significant difference (*p* < 0.01).

**Table 3:** The hydrolysis conditions for different proteases.

Proteases	Temperature (°C)	pH
Papain	50	7
Trypsin	37	8
Alkaline	55	8

**Table 4:** Design of experiments—factor level table.

Parameters	Coded Level		
	−1	0	1
A Temperature (°C)	55	60	65
B Time (h)	1.5	2	2.5
C E/S (U/g)	6000	8000	10,000

## Data Availability

The data presented in this study are available upon request from the corresponding author. The data are not publicly available due to privacy.
